# Supplementary material for: Multi‐dataset identification of innovative feature genes and molecular mechanisms in keratoconus
Source: J Cell Mol Med. 2024 Sep 19;28(18):e70079. doi: 10.1111/jcmm.70079 (PMC11412914; doi:10.1111/jcmm.70079)
Supplement: Supplementary file 2 — Material S2. [file JCMM-28-e70079-s002.zip › 7.GSEA analysis/2.ATOH7/KEGG_INSULIN_SIGNALING_PATHWAY.html]

Details for gene set KEGG\_INSULIN\_SIGNALING\_PATHWAY[GSEA]

|  || Dataset | ATOH7.group.cls#h\_versus\_l |
| Phenotype | group.cls#h\_versus\_l |
| Upregulated in class | h |
| GeneSet | KEGG\_INSULIN\_SIGNALING\_PATHWAY |
| Enrichment Score (ES) | 0.35277864 |
| Normalized Enrichment Score (NES) | 1.5227747 |
| Nominal p-value | 0.04771372 |
| FDR q-value | 0.2851275 |
| FWER p-Value | 0.743 |
Table: GSEA Results Summary

  

Fig 1: Enrichment plot: KEGG\_INSULIN\_SIGNALING\_PATHWAY      
 Profile of the Running ES Score & Positions of GeneSet Members on the Rank Ordered List

  

| SYMBOL | TITLE | RANK IN GENE LIST | RANK METRIC SCORE | RUNNING ES | CORE ENRICHMENT || 1 | AKT3 | na | 9 | 1.043 | 0.0657 | Yes |
| 2 | IRS1 | na | 55 | 0.848 | 0.1179 | Yes |
| 3 | SOCS2 | na | 89 | 0.782 | 0.1664 | Yes |
| 4 | SHC2 | na | 290 | 0.628 | 0.2003 | Yes |
| 5 | PRKACB | na | 829 | 0.474 | 0.2147 | Yes |
| 6 | PPP1R3B | na | 1266 | 0.412 | 0.2281 | Yes |
| 7 | GCK | na | 1752 | 0.364 | 0.2371 | Yes |
| 8 | PRKACG | na | 1784 | 0.361 | 0.2590 | Yes |
| 9 | PRKAA2 | na | 2242 | 0.326 | 0.2664 | Yes |
| 10 | PPP1R3A | na | 2330 | 0.321 | 0.2842 | Yes |
| 11 | FBP2 | na | 2527 | 0.307 | 0.2979 | Yes |
| 12 | PCK2 | na | 2919 | 0.285 | 0.3046 | Yes |
| 13 | CALML6 | na | 3497 | 0.256 | 0.3041 | Yes |
| 14 | IRS2 | na | 3662 | 0.249 | 0.3151 | Yes |
| 15 | SLC2A4 | na | 4197 | 0.229 | 0.3142 | Yes |
| 16 | MAP2K1 | na | 4240 | 0.228 | 0.3273 | Yes |
| 17 | HK2 | na | 5782 | 0.177 | 0.2940 | Yes |
| 18 | CRKL | na | 5867 | 0.175 | 0.3026 | Yes |
| 19 | PHKG2 | na | 5869 | 0.175 | 0.3136 | Yes |
| 20 | SOCS3 | na | 5926 | 0.173 | 0.3229 | Yes |
| 21 | EIF4E1B | na | 6119 | 0.168 | 0.3279 | Yes |
| 22 | PIK3R3 | na | 6166 | 0.166 | 0.3371 | Yes |
| 23 | PPARGC1A | na | 6498 | 0.157 | 0.3375 | Yes |
| 24 | EIF4EBP1 | na | 6778 | 0.149 | 0.3388 | Yes |
| 25 | TSC1 | na | 6924 | 0.145 | 0.3438 | Yes |
| 26 | PCK1 | na | 6929 | 0.145 | 0.3528 | Yes |
| 27 | RAPGEF1 | na | 7417 | 0.132 | 0.3471 | No |
| 28 | NRAS | na | 7693 | 0.126 | 0.3471 | No |
| 29 | G6PC2 | na | 8689 | 0.105 | 0.3249 | No |
| 30 | CRK | na | 8997 | 0.099 | 0.3223 | No |
| 31 | SOCS1 | na | 9819 | 0.084 | 0.3039 | No |
| 32 | RHOQ | na | 10046 | 0.081 | 0.3024 | No |
| 33 | CALM3 | na | 10070 | 0.080 | 0.3068 | No |
| 34 | ARAF | na | 10150 | 0.079 | 0.3095 | No |
| 35 | GRB2 | na | 10441 | 0.075 | 0.3059 | No |
| 36 | SHC4 | na | 10680 | 0.071 | 0.3035 | No |
| 37 | HK1 | na | 11153 | 0.064 | 0.2938 | No |
| 38 | INS | na | 11499 | 0.059 | 0.2876 | No |
| 39 | SH2B2 | na | 11701 | 0.056 | 0.2854 | No |
| 40 | PRKAB2 | na | 11761 | 0.056 | 0.2872 | No |
| 41 | TRIP10 | na | 11771 | 0.055 | 0.2904 | No |
| 42 | PHKA2 | na | 11792 | 0.055 | 0.2933 | No |
| 43 | EIF4E | na | 12069 | 0.052 | 0.2886 | No |
| 44 | PRKACA | na | 12347 | 0.048 | 0.2836 | No |
| 45 | PRKX | na | 12697 | 0.044 | 0.2763 | No |
| 46 | PRKAB1 | na | 12927 | 0.041 | 0.2723 | No |
| 47 | HK3 | na | 13130 | 0.039 | 0.2689 | No |
| 48 | PPP1CC | na | 14047 | 0.030 | 0.2443 | No |
| 49 | MAPK1 | na | 14536 | 0.025 | 0.2318 | No |
| 50 | HRAS | na | 14559 | 0.025 | 0.2328 | No |
| 51 | SHC1 | na | 15160 | 0.020 | 0.2167 | No |
| 52 | RAF1 | na | 15219 | 0.019 | 0.2162 | No |
| 53 | EXOC7 | na | 15613 | 0.016 | 0.2059 | No |
| 54 | AKT1 | na | 15787 | 0.014 | 0.2018 | No |
| 55 | GSK3B | na | 16028 | 0.013 | 0.1957 | No |
| 56 | PIK3CD | na | 16202 | 0.011 | 0.1914 | No |
| 57 | FLOT1 | na | 16216 | 0.011 | 0.1917 | No |
| 58 | CALM1 | na | 16251 | 0.011 | 0.1914 | No |
| 59 | LIPE | na | 16627 | 0.008 | 0.1810 | No |
| 60 | SORBS1 | na | 16752 | 0.007 | 0.1779 | No |
| 61 | PRKAG1 | na | 16803 | 0.006 | 0.1768 | No |
| 62 | MAPK3 | na | 16821 | 0.006 | 0.1767 | No |
| 63 | PYGL | na | 16845 | 0.006 | 0.1764 | No |
| 64 | MAPK8 | na | 17140 | 0.003 | 0.1681 | No |
| 65 | PRKAG3 | na | 17215 | 0.003 | 0.1662 | No |
| 66 | BAD | na | 17218 | 0.002 | 0.1663 | No |
| 67 | PPP1CA | na | 17263 | 0.002 | 0.1651 | No |
| 68 | PPP1R3C | na | 17401 | 0.001 | 0.1612 | No |
| 69 | EIF4E2 | na | 17836 | -0.003 | 0.1488 | No |
| 70 | ACACB | na | 17889 | -0.003 | 0.1475 | No |
| 71 | SOCS4 | na | 18050 | -0.004 | 0.1432 | No |
| 72 | PRKAR2A | na | 18083 | -0.005 | 0.1426 | No |
| 73 | PTPN1 | na | 18450 | -0.008 | 0.1325 | No |
| 74 | PRKAA1 | na | 18507 | -0.009 | 0.1314 | No |
| 75 | PRKAR1A | na | 18611 | -0.010 | 0.1290 | No |
| 76 | RHEB | na | 18644 | -0.010 | 0.1287 | No |
| 77 | INSR | na | 18814 | -0.011 | 0.1246 | No |
| 78 | PHKA1 | na | 18871 | -0.012 | 0.1237 | No |
| 79 | INPP5K | na | 18962 | -0.013 | 0.1219 | No |
| 80 | RPS6 | na | 18999 | -0.013 | 0.1217 | No |
| 81 | PRKCI | na | 19141 | -0.014 | 0.1185 | No |
| 82 | PPP1R3D | na | 19388 | -0.016 | 0.1124 | No |
| 83 | SREBF1 | na | 19439 | -0.017 | 0.1120 | No |
| 84 | PPP1CB | na | 19627 | -0.018 | 0.1077 | No |
| 85 | FOXO1 | na | 19979 | -0.022 | 0.0990 | No |
| 86 | CBLC | na | 20019 | -0.022 | 0.0992 | No |
| 87 | RPTOR | na | 20087 | -0.023 | 0.0987 | No |
| 88 | GYS2 | na | 20088 | -0.023 | 0.1001 | No |
| 89 | CALML3 | na | 20596 | -0.028 | 0.0872 | No |
| 90 | MAP2K2 | na | 20653 | -0.028 | 0.0874 | No |
| 91 | PIK3CB | na | 20685 | -0.028 | 0.0883 | No |
| 92 | MAPK10 | na | 20989 | -0.032 | 0.0815 | No |
| 93 | PRKCZ | na | 21192 | -0.034 | 0.0778 | No |
| 94 | PHKB | na | 21278 | -0.035 | 0.0775 | No |
| 95 | PIK3CA | na | 21359 | -0.036 | 0.0775 | No |
| 96 | PTPRF | na | 21634 | -0.039 | 0.0720 | No |
| 97 | MKNK2 | na | 21890 | -0.042 | 0.0673 | No |
| 98 | CALM2 | na | 21949 | -0.042 | 0.0683 | No |
| 99 | PIK3R2 | na | 22209 | -0.045 | 0.0636 | No |
| 100 | PRKAG2 | na | 22217 | -0.045 | 0.0663 | No |
| 101 | PDE3A | na | 22259 | -0.046 | 0.0680 | No |
| 102 | PRKAR1B | na | 22541 | -0.049 | 0.0629 | No |
| 103 | SOS2 | na | 22550 | -0.049 | 0.0658 | No |
| 104 | RPS6KB1 | na | 22694 | -0.051 | 0.0649 | No |
| 105 | SHC3 | na | 22782 | -0.052 | 0.0656 | No |
| 106 | RPS6KB2 | na | 23175 | -0.057 | 0.0579 | No |
| 107 | PYGB | na | 23193 | -0.057 | 0.0610 | No |
| 108 | IRS4 | na | 23255 | -0.058 | 0.0630 | No |
| 109 | IKBKB | na | 23356 | -0.060 | 0.0639 | No |
| 110 | FLOT2 | na | 23622 | -0.064 | 0.0602 | No |
| 111 | PDPK1 | na | 24308 | -0.074 | 0.0451 | No |
| 112 | CBL | na | 24630 | -0.079 | 0.0409 | No |
| 113 | MKNK1 | na | 24663 | -0.080 | 0.0450 | No |
| 114 | MAPK9 | na | 24826 | -0.082 | 0.0455 | No |
| 115 | ACACA | na | 24975 | -0.085 | 0.0466 | No |
| 116 | BRAF | na | 25048 | -0.086 | 0.0500 | No |
| 117 | KRAS | na | 25083 | -0.087 | 0.0544 | No |
| 118 | MTOR | na | 25211 | -0.089 | 0.0564 | No |
| 119 | GYS1 | na | 25673 | -0.098 | 0.0492 | No |
| 120 | FBP1 | na | 25972 | -0.103 | 0.0472 | No |
| 121 | PRKAR2B | na | 26006 | -0.104 | 0.0528 | No |
| 122 | PIK3CG | na | 26210 | -0.109 | 0.0538 | No |
| 123 | PHKG1 | na | 26386 | -0.112 | 0.0558 | No |
| 124 | SOS1 | na | 26852 | -0.122 | 0.0501 | No |
| 125 | AKT2 | na | 27111 | -0.128 | 0.0507 | No |
| 126 | PYGM | na | 27636 | -0.142 | 0.0445 | No |
| 127 | PIK3R1 | na | 27720 | -0.144 | 0.0512 | No |
| 128 | FASN | na | 28506 | -0.164 | 0.0389 | No |
| 129 | INPP5D | na | 28637 | -0.168 | 0.0458 | No |
| 130 | TSC2 | na | 29366 | -0.190 | 0.0367 | No |
| 131 | CALML5 | na | 29931 | -0.208 | 0.0336 | No |
| 132 | PKLR | na | 30222 | -0.219 | 0.0390 | No |
| 133 | ELK1 | na | 30931 | -0.244 | 0.0340 | No |
| 134 | CBLB | na | 31517 | -0.270 | 0.0341 | No |
| 135 | PDE3B | na | 32353 | -0.316 | 0.0300 | No |
| 136 | PIK3R5 | na | 34383 | -0.611 | 0.0100 | No |
Table: GSEA details [plain text format]

  

Fig 2: KEGG\_INSULIN\_SIGNALING\_PATHWAY      
 Blue-Pink O' Gram in the Space of the Analyzed GeneSet

  

Fig 3: KEGG\_INSULIN\_SIGNALING\_PATHWAY: Random ES distribution      
 Gene set null distribution of ES for **KEGG\_INSULIN\_SIGNALING\_PATHWAY**
